# The role of circulating galectin-1 in type 2 diabetes and chronic kidney disease: evidence from cross-sectional, longitudinal and Mendelian randomisation analyses

**DOI:** 10.1007/s00125-021-05594-1

**Published:** 2021-11-07

**Authors:** Isabel Drake, Emanuel Fryk, Lena Strindberg, Annika Lundqvist, Anders H. Rosengren, Leif Groop, Emma Ahlqvist, Jan Borén, Marju Orho-Melander, Per-Anders Jansson

**Affiliations:** 1 Department of Clinical Sciences in Malmö, Lund University Diabetes Centre, Lund University, Malmö, Sweden; 2 Department of Molecular and Clinical Medicine, Institute of Medicine, Sahlgrenska Academy, University of Gothenburg, Gothenburg, Sweden; 3 Department of Neuroscience and Physiology, Sahlgrenska Academy, University of Gothenburg, Gothenburg, Sweden

**Keywords:** ANDIS, Chronic kidney disease, Galectin-1, Human, Malmö Diet Cancer, Mendelian randomisation, Population-based, Prospective, Type 2 diabetes

## Abstract

**Aims/hypothesis:**

Galectin-1 modulates inflammation and angiogenesis, and cross-sectional studies indicate that galectin-1 may be a uniting factor between obesity, type 2 diabetes and kidney function. We examined whether circulating galectin-1 can predict incidence of chronic kidney disease (CKD) and type 2 diabetes in a middle-aged population, and if Mendelian randomisation (MR) can provide evidence for causal direction of effects.

**Methods:**

Participants (*n* = 4022; 58.6% women) in the Malmö Diet and Cancer Study–Cardiovascular Cohort enrolled between 1991 and 1994 (mean age 57.6 years) were examined. eGFR was calculated at baseline and after a mean follow-up of 16.6 ± 1.5 years. Diabetes status was ascertained through registry linkage (mean follow-up of 18.4 ± 6.1 years). The associations of baseline galectin-1 with incident CKD and type 2 diabetes were assessed with Cox regression, adjusting for established risk factors. In addition, a genome-wide association study on galectin-1 was performed to identify genetic instruments for two-sample MR analyses utilising the genetic associations obtained from the Chronic Kidney Disease Genetics (CKDGen) Consortium (41,395 cases and 439,303 controls) and the DIAbetes Genetics Replication And Meta-analysis (DIAGRAM) consortium (74,124 cases and 824,006 controls). One genome-wide significant locus in the galectin-1 gene region was identified (sentinel SNP rs7285699; *p* = 2.4 × 10^−11^). The association between galectin-1 and eGFR was also examined in individuals with newly diagnosed diabetes from the All New Diabetics In Scania (ANDIS) cohort.

**Results:**

Galectin-1 was strongly associated with lower eGFR at baseline (*p* = 2.3 × 10^−89^) but not with incident CKD. However, galectin-1 was associated with increased risk of type 2 diabetes (per SD increase, HR 1.12; 95% CI 1.02, 1.24). Two-sample MR analyses could not ascertain a causal effect of galectin-1 on CKD (OR 0.92; 95% CI 0.82, 1.02) or type 2 diabetes (OR 1.05; 95% CI 0.98, 1.14) in a general population. However, in individuals with type 2 diabetes from ANDIS who belonged to the severe insulin-resistant diabetes subgroup and were at high risk of diabetic nephropathy, genetically elevated galectin-1 was significantly associated with higher eGFR (*p* = 5.7 × 10^−3^).

**Conclusions/interpretation:**

Galectin-1 is strongly associated with lower kidney function in cross-sectional analyses, and two-sample MR analyses suggest a causal protective effect on kidney function among individuals with type 2 diabetes at high risk of diabetic nephropathy. Future studies are needed to explore the mechanisms by which galectin-1 affects kidney function and whether it could be a useful target among individuals with type 2 diabetes for renal improvement.

**Graphical abstract:**

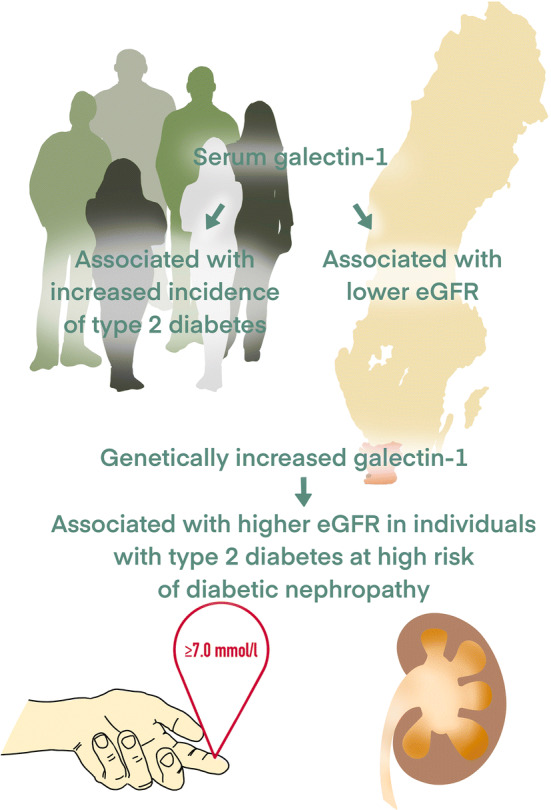

**Supplementary Information:**

The online version contains peer-reviewed but unedited supplementary material available at 10.1007/s00125-021-05594-1.

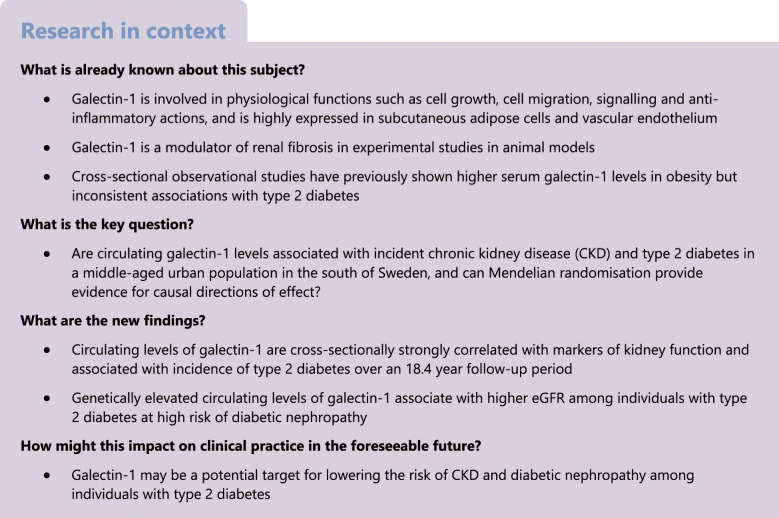



## Introduction

Diabetes is a global health concern, currently affecting over 400 million people worldwide [1]. Half of all individuals with type 2 diabetes also develop chronic kidney disease (CKD), with significant implications on daily life and medical costs [2]. Recently, Ahlqvist et al disentangled the complex diabetes phenotype in a data-driven cluster analysis to identify subgroups of patients for optimisation of treatment and lowering of complication risk. The risk of kidney complications was markedly increased in individuals with type 2 diabetes with severe insulin-resistant diabetes (SIRD) and the authors argued that it might be possible to refine the stratification further through inclusion of additional cluster variables such as other biomarkers [3].

In recent years, there has been a growing interest in the role of galectins in human disease [4, 5]. Galectin-1 is a highly conserved carbohydrate-binding protein involved in cell growth, with anti-inflammatory effects reported in several studies [4, 6]. Experimental data suggest that galectin-1 may modulate kidney fibrosis in mouse models of diabetes [7]. In human studies, increased galectin-1 expression was reported in renal biopsy samples from individuals with diabetic kidney disease, and silencing of galectin-1 in podocytes preserved the phenotype during high-glucose stress [8]. Furthermore, measured circulating galectin-1 was an independent predictor of renal function decline in patients undergoing coronary angiography [9]. However, other studies have reported renal protective effects of galectin-1 via anti-inflammatory mechanisms [10]. Pre-treatment with galectin-1 attenuated the influx of proinflammatory cytokines and renal cell injury in an ischaemia–reperfusion injury rat model, and exogenous addition of galectin-1 decreased proinflammatory cytokine release from the renal tubular epithelial cells.

Galectin-1 is highly expressed in adipose tissue and vascular endothelium [11, 12]. Specifically, galectin-1 acts through the endothelial surface receptor neuropilin-1, a vascular endothelial growth factor B (VEGF-B) co-receptor that has been suggested to regulate lipid uptake over the vascular wall [13–15]. Interestingly, in previous studies, we have measured higher galectin-1 levels in the subcutaneous interstitial fluid, but similar serum levels, in individuals with type 2 diabetes compared with healthy control participants [16]. We have also observed a strong cross-sectional association between circulating levels of galectin-1 and several markers of the metabolic syndrome in a population-based cohort, but BMI-adjusted galectin-1 levels were lower in individuals with type 2 diabetes [17]. However, longitudinal evidence on the association between galectin-1 and diabetes risk remains to be shown.

Using the large, population-based Malmö Diet and Cancer Study–Cardiovascular Cohort (MDCS-CC), we aimed to examine whether circulating galectin-1 levels associate with incident CKD and type 2 diabetes, independently of established risk factors. In addition, we adopted two-sample Mendelian randomisation (MR) analyses to assess the causal nature of such associations.

## Methods

### The Malmö Diet and Cancer Study

**Fig. 1:**
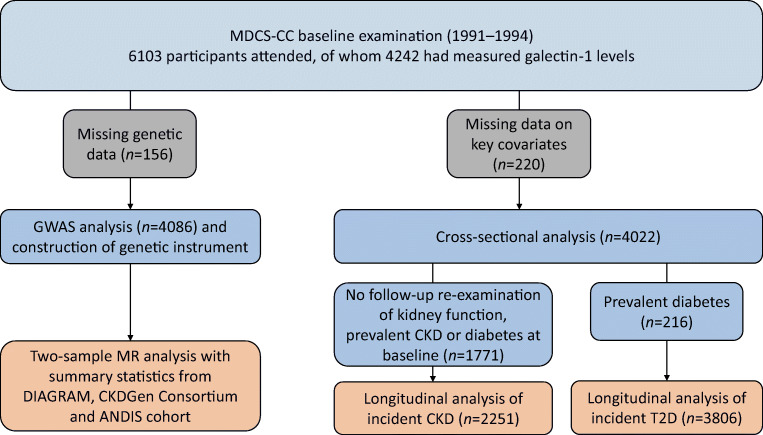
A flow chart of participants of the MDCS-CC with circulating levels of galectin-1 who were included in the cross-sectional and longitudinal analyses and in GWAS and MR analyses.T2D, type 2 diabetes

The Malmö Diet and Cancer Study (MDCS) is a population-based prospective cohort study established between 1991 and 1996 in Sweden. Detailed descriptions of the cohort and representability have been published previously [18]. Between October 1991 and February 1994, every other MDCS participant was invited to join a sub-study on cardiovascular disease risk (MDCS-CC; *N* = 6103) [19]. The population samples used for analyses are shown in Fig. 1. Baseline blood samples were available from 4242 participants, of whom 220 individuals had missing data for covariates, leaving 4022 participants for descriptive analysis (mean age 57.6 ± 6.0 years; 2355 women and 1667 men). The MDCS was approved by the Ethics Committee at Lund University (Malmö, Sweden; LU 51–90, LU 204–00 and Dnr. 469/2006), and written, informed consent was obtained from all participants in accordance with the Declaration of Helsinki.

### MDCS-CC baseline measurements

Detailed descriptions of clinical characteristics and standard anthropometric and blood-based measurements can be found in the electronic supplementary material (ESM) Methods. Participants completed an extensive baseline questionnaire including questions on lifestyle, socioeconomic factors and medical history. Direct measurements included height (cm) and weight (kg), which were used to calculate BMI (kg/m^2^). Blood pressure (mmHg) was measured after 5 min of supine rest. Blood samples were donated at baseline after an overnight fast. Plasma creatinine (μmol/l) levels were measured and analysed with the Jaffé method [20]. eGFR was calculated based on the previously reported Chronic Kidney Disease Epidemiology Collaboration 2009 creatinine-based equation [21]. A factor of 0.0113 was included to convert creatinine levels measured in μmol/l into mg/dl.

### Galectin-1 measurements

Galectin-1 was measured in human sera sampled at the study baseline with the Human Galectin-1 Quantikine ELISA Kit (R&D Systems, MN, USA) according to the manufacturer’s instructions. Intra-assay and inter-assay coefficients of variation were 7.1% and 9.5%, respectively.

### Incidence of CKD, type 2 diabetes and secondary outcomes

Incidence of CKD was defined as having an eGFR <60 ml min^−1^ [1.73 m]^−2^ at the follow-up re-examination between 2007 and 2012 (mean follow-up of 16.6 ± 1.5 years) [22]. Diabetes status at baseline and during follow-up was ascertained through linkage to regional and national registries until 31 December 2014 and through the baseline screening (mean follow-up of 18.4 ± 6.1 years). Secondary outcomes included coronary artery disease and all-cause and cause-specific mortality. A more detailed description of the ascertainment of all outcomes is found in the ESM Methods: ‘Type 2 diabetes ascertainment’ and ‘Secondary outcomes’.

### Genetic analyses and selection of instrumental variables

**Fig. 2:**
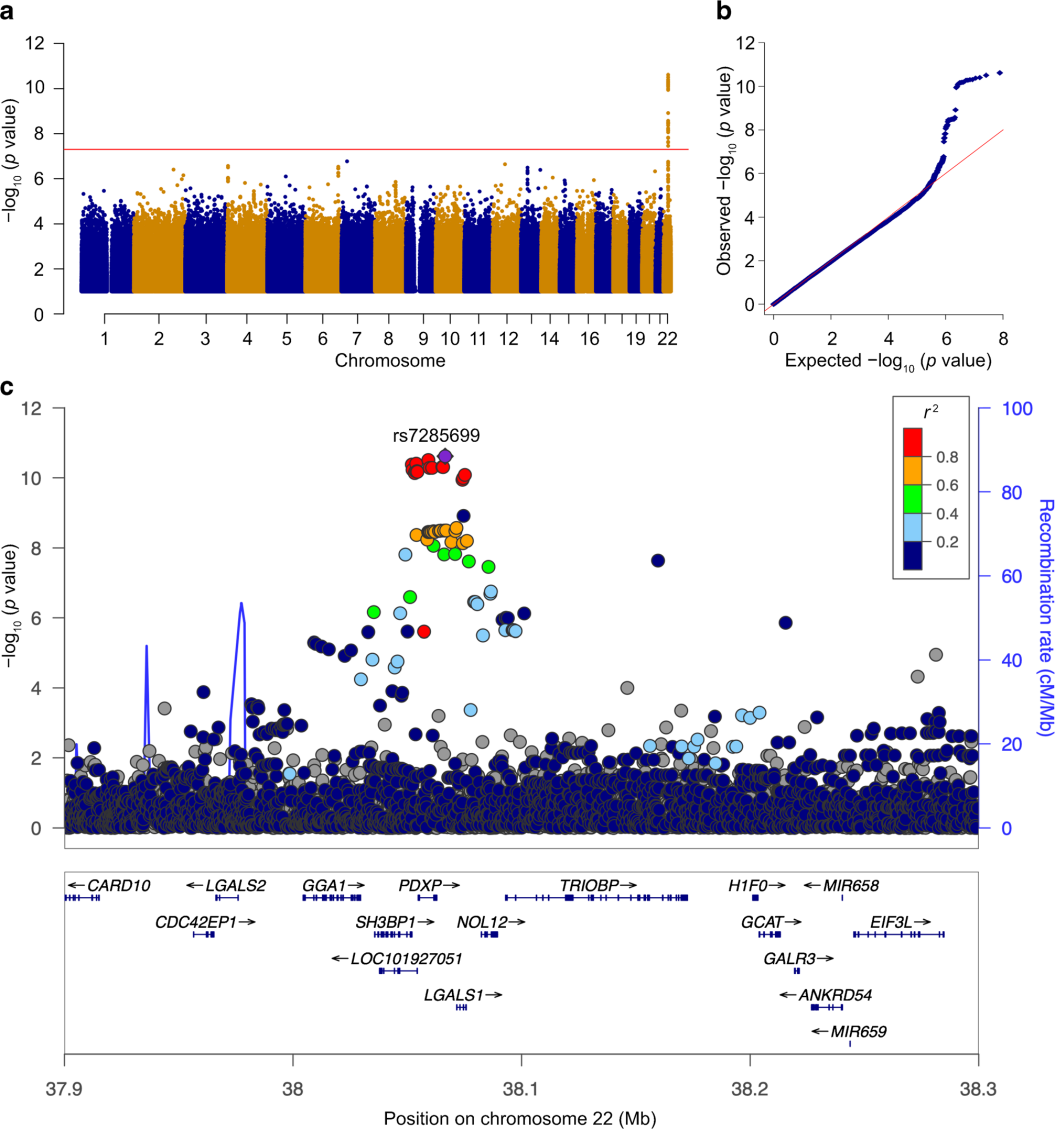
Genome-wide analyses for fasting serum concentration of galectin-1 in the MDCS-CC. (**a**) Manhattan plot of SNPs with *p* values <0.10 based on genome-wide analysis (chromosomes 1–22) in *n* = 4086 participants from the MDCS-CC. Red line indicates the genome-wide significant *p* value of 5 × 10^−8^. (**b**) Quantile–quantile (QQ) plot. (**c**) Regional locus zoom plot of associations at/near the galectin-1 gene (*LGALS1*). The purple diamond indicates the sentinel SNP (rs7285699; *p* = 2.4 × 10^−11^), and all identified SNPs within different degrees of perfect linkage disequilibrium are also shown (*r*^2^ ≥ 0.80 [red], <0.8–0.6 [orange], <0.6–0.4 [green], <0.4–0.2 [light blue] and ≤0.2 [dark blue]) at this locus. cM, centimorgans; Mb, megabase

Genotyping was performed using Illumina HumanOmniExpress BeadChip v. 1 (Illumina, CA, USA) at Broad Institute, MA, USA, and the dataset was imputed to the 1000 Genomes reference panel (phase 1, version 3) after standard quality control procedures (for more details, see ESM Methods ‘Genotyping quality control’). Galectin-1 levels were natural log (log_*e*_)-normalised and adjusted for age, age^2^, sex and the first three principal components of ancestry from multi-dimensional scaling in a linear regression model. Age^2^ adjustment was included to account for non-linear age effects. The residuals were rank inverse normalised and used as the phenotype for association testing. PLINK (version 1.9; http://pngu.mgh.harvard.edu/~purcell/plink/, available from 15 May 2014) was used to fit linear regression models using an additive genetic model [23]. Results are presented using a Manhattan plot and a locus zoom plot of the galectin-1 gene region (*LGALS1*) (Fig. 2). The Q–Q plot and the genomic inflation factor (λ) were used to assess goodness of fit. The galectin-1 gene region was defined using Ensemble BioMart (GRCh37 Build). SNPs within 300 kb of the galectin-1 gene (*LGALS1*) were assessed for inclusion as genetic instruments. The sentinel SNP (rs7285699) was defined as the SNP with the lowest *p* value at the genome-wide significant locus. Two additional independent variants were identified using a stepwise conditional analysis with a conditional *p* value threshold of 0.01 (see ESM Methods ‘Construction of multi-SNP instrument’). Details of the variants included as genetic instruments can be found in ESM Table 1.

### The All New Diabetics In Scania (ANDIS) study

ANDIS is an ongoing study that aims to include all individuals with newly diagnosed diabetes mellitus in the south of Sweden (the Scania region). Using this study, it was recently suggested that diabetes mellitus can be clustered into five subgroups. One of the identified clusters corresponded to the classical type 1 diabetes phenotype, and individuals with type 2 diabetes could be further sub-stratified into four clusters: severe insulin-deficient diabetes (SIDD); mild obesity-related diabetes (MOD); mild age-related diabetes (MARD); and SIRD [3]. Between 1 February 2007 and 30 September 2016, 9367 participants were enrolled and had eGFR and genome-wide association study (GWAS) genotyping data measured. In this group, 1116 participants were clustered as SIRD. Within each of the four type 2 diabetes clusters, 44 participants had data on plasma galectin-1 levels (arbitrary units [AU]) quantified using the Olink Target 96 Immuno-Oncology proximity extension assay (Olink Proteomics, Sweden) and analysed at SciLife Laboratory (Uppsala, Sweden). The mean levels of galectin-1 across the four type 2 diabetes clusters were examined, and differences were tested using ANOVA and linear regression with adjustment for age, sex and BMI. Further, the association between galectin-1 and eGFR at the time of diabetes diagnosis was assessed using linear regression, adjusting for age, sex, BMI, HOMA2-IR (calculator downloaded 10 December 2016 [24]), HOMA2-B and HbA_1c_ (all measured at time of diagnosis). The sentinel SNP from the galectin-1 GWAS in the MDCS-CC (rs7285699) was examined in relation to eGFR among all individuals with type 2 diabetes and within the specific clusters.

### Statistical analyses

We examined the baseline characteristics of participants across quartiles of baseline galectin-1 levels. A linear regression model was used to test differences in galectin-1 levels by baseline characteristics, adjusting for age, sex and BMI. Longitudinal change in eGFR (absolute change and annual relative change) per SD increase in galectin-1 was examined using a linear regression model. The risk of incident CKD at the re-examination was assessed using a Cox regression model with follow-up time until the date of re-examination as the timescale. Longitudinal associations of baseline galectin-1 levels in relation to incidence of type 2 diabetes as well as secondary outcomes were examined using Cox proportional hazards regression with follow-up time until 31 December 2014 as the timescale. We estimated HRs and 95% CIs per quartile of galectin-1 using the first quartile as reference, as well as the HR per SD increase in galectin-1 levels. No deviations from linearity were detected based on fitting restricted cubic splines and testing for non-linearity using the likelihood ratio test. The proportional hazards assumption was fulfilled for all presented models based on the Schoenfeld residuals test. All covariates were selected a priori and included established risk factors for CKD and type 2 diabetes, respectively. To assess the discriminative usefulness of galectin-1 in addition to established risk factors, we calculated Harell’s concordance index and category-free net reclassification improvement (cNRI). Differences between models with and without galectin-1 were assessed using the likelihood ratio test.

We performed two-sample MR by using previously published summary statistics of genetic associations with outcomes. Genetic associations with CKD and eGFR were obtained from the Chronic Kidney Disease Genetics (CKDGen) Consortium meta-analysis (41,395 cases and 439,303 control participants with European ancestry) [25]. Further, we examined the association between galectin-1 and eGFR stratified by diabetes mellitus status in the CKDGen Consortium study based on summary statistics from Pattaro et al [26]. Genetic associations with type 2 diabetes were obtained by including summary statistics from the DIAbetes Genetics Replication And Meta-analysis (DIAGRAM) consortium [27]. The meta-analysis included 32 GWAS of type 2 diabetes (cases = 74,124, control participants = 824,006 with European ancestry). We used the BMI-unadjusted results to avoid collider bias. The causal effects of galectin-1 on the examined outcomes were assessed using the sentinel SNP (rs7285699) as the instrumental variable and estimated using the Wald ratio method. For CKD and type 2 diabetes, we further estimated causal effects using the fixed effect inverse variance-weighted (IVW) method, including three variants associated with galectin-1 at *p* < 0.01 after stepwise conditional and joint analysis.

All tests were two-sided, and statistical significance level was set at *p* < 0.025 after Bonferroni correction to account for the two main outcomes examined. All analyses were performed using Stata version 14.2 (TX, USA), SPSS Statistics version 25 (IBM, New York, NY, USA) and the statistical program R version 3.6.0 (R Foundation for Statistical Computing, Vienna, Austria; www.R-project.org, available from 26 April 2019). MR analyses were performed using the ‘TwoSample MR’ package [28].

## Results

### Clinical characteristics of the MDCS-CC by galectin-1 levels

Clinical characteristics of the MDCS-CC participants are shown in Table 1. After adjustment for age and sex, higher galectin-1 levels were strongly associated with higher baseline BMI (*p* = 9.0 × 10^−54^) and lower eGFR (*p* = 2.3 × 10^−89^; Table 1). Galectin-1 also showed positive associations with fasting insulin (*p* = 6.6 × 10^−22^), triacylglycerol (*p* = 1.5 × 10^−33^) and high-sensitivity C-reactive protein (hsCRP) (*p* = 2.7 × 10^−25^) and significant associations with HbA_1c_ (*p* = 5.5 × 10^−3^) and HOMA-IR (*p* = 6.3 × 10^−19^; Table 1). Associations were robust after additional adjustment for BMI, with the exception of HOMA-IR and HbA_1c_. Participants with a parental history of diabetes mellitus had lower levels of galectin-1, and similarly, participants with prevalent diabetes mellitus also had lower galectin-1 levels after adjustment for age, sex and BMI.

**Table 1:** Baseline clinical characteristics by quartiles of galectin-1 levels among participants in the MDCS-CC (*n* = 4022)

Characteristic	Quartiles of galectin-1 levels				*p* value^a^	*p* value^b^
	Q1	Q2	Q3	Q4		
Number of participants	1006	1005	1006	1005		
Galectin-1, ng/ml	16.8 (14.7, 18.2)	22.1 (20.9, 23.3)	27.6 (25.8, 29.3)	36.1 (33.3, 41.4)	–^c^	–^c^
Age, years	55.9 (51.1, 61.2)	57.3 (51.7, 62.4)	58.6 (53.3, 63.3)	60.0 (54.3, 64.1)	9.1 × 10^−31^	2.1 × 10^−24^
Male sex, %	39.5	40.2	41.1	45.1	0.092	0.721
University or college degree, %	13.0	13.0	11.8	8.9	0.183	0.751
Current smoking, %	26.8	26.9	27.1	24.8	0.559	0.058
Prevalent diabetes, %	7.0	4.4	3.7	6.5	0.206	1.1 × 10^−3^
Prevalent CKD, %	0.5	0.4	1.3	5.0	1.1 × 10^−14^	1.2 × 10^−13^
Parental history of diabetes, %	1.6	0.5	0.8	0.6	0.026	0.018
Use of lipid-lowering drugs, %	1.4	2.3	2.4	3.3	8.1 × 10^−3^	0.039
Use of anti-hypertensives, %	11.5	14.8	17.8	24.5	8.9 × 10^−10^	1.3 × 10^−3^
BMI, kg/m^2^	24.0 (22.0, 26.5)	24.7 (22.7, 27.2)	25.6 (23.4, 27.8)	26.6 (24.2, 29.5)	9.0 × 10^−54^	–
Systolic blood pressure, mmHg	136 (122, 150)	140 (128, 150)	140 (130, 152)	142 (130, 160)	1.9 × 10^−8^	0.01
Diastolic blood pressure, mmHg	85 (80, 90)	86 (80, 92)	86 (80, 92)	88 (80, 95)	4.3 × 10^−6^	0.366
Fasting glucose, mmol/l	4.8 (4.6, 5.2)	4.9 (4.6, 5.2)	4.9 (4.6, 5.3)	5.0 (4.7, 5.5)	0.030	0.026
Fasting insulin, pmol/l^d^	36 (24, 48)	36 (24, 54)	42 (24, 60)	48 (30, 66)	6.6 × 10^−22^	7.3 × 10^−3^
HbA_1c_, mmol/mol	40 (36, 43)	40 (36, 43)	41 (37, 43)	41 (37, 44)	5.5 × 10^−3^	0.949
HbA_1c_, % (Mono-S)^e^	4.8 (4.5, 5.1)	4.8 (4.5, 5.1)	4.9 (4.6, 5.1)	4.9 (4.6, 5.2)	5.5 × 10^−3^	0.949
Serum triacylglycerols, mmol/l	1.0 (0.8, 1.4)	1.1 (0.8, 1.5)	1.2 (0.9, 1.7)	1.3 (1.0, 1.8)	1.5 × 10^−33^	4.5 × 10^−14^
LDL-cholesterol, mmol/l	3.9 (3.3, 4.6)	4.1 (3.5, 4.7)	4.2 (3.5, 4.8)	4.2 (3.6, 4.9)	3.6 × 10^−10^	4.9 × 10^−7^
HDL-cholesterol, mmol/l	1.4 (1.2, 1.7)	1.4 (1.1, 1.6)	1.4 (1.1, 1.6)	1.3 (1.1, 1.5)	2.3 × 10^−12^	0.012
hsCRP, mg/l	1.0 (0.5, 2.0)	1.2 (0.6, 2.5)	1.4 (0.7, 2.9)	1.8 (0.9, 3.7)	2.7 × 10^−25^	7.8 × 10^−10^
HOMA-IR^f^	1.2 (0.8, 1.9)	1.3 (0.8, 2.0)	1.5 (0.9, 2.3)	1.7 (1.1, 2.6)	6.3 × 10^−19^	0.099
Cystatin C, mg/l	0.71 (0.64, 0.78)	0.75 (0.68, 0.83)	0.78 (0.71, 0.85)	0.81 (0.73, 0.91)	5.0 × 10^−78^	2.4 × 10^−64^
Creatinine, μmol/l	78 (70, 88)	82 (74, 90)	84 (75, 93)	87 (78, 97)	9.1 × 10^−48^	8.5 × 10^−50^
eGFR, ml min^−1^ [1.73 m]^−2^	96.0 (87.6, 103.4)	90.7 (82.2, 99.0)	87.9 (79.6, 96.3)	84.6 (74.8, 93.7)	2.3 × 10^−89^	1.2 × 10^−81^

Data are presented as median (IQR) for continuous variables and as percentage for categorical variables

^a^*p* value from linear regression model using log_*e*_-transformed galectin-1 levels as the dependent variable and clinical characteristic as the independent variable, adjusting for age and sex

^b^*p* value from linear regression model using log_*e*_-transformed galectin-1 levels as the dependent variable and clinical characteristic as the independent variable, adjusting for age, sex and BMI

^c^Statistical comparison was not performed for galectin-1 levels, as quartile data for this measurement was used to define the groups

^d^Converted from mU/l with a conversion factor of 1.0 mU/l = 6.0 pmol/l

^e^Original analysis method: Mono-S was the standard method for HbA_1c_ analysis in Sweden at the time of study baseline; normal range, 3.9–5.3% [40]

^f^HOMA-IR calculated according to Matthews et al [41]

### Galectin-1 and incident CKD and type 2 diabetes in the MDCS-CC

Participants with high galectin-1 levels at baseline had lower eGFR at both baseline and follow-up and a non-significant smaller absolute decline, compared with those with low galectin-1 levels. There was further a significant tendency for a smaller mean annual relative decline (*p* = 5.6 × 10^−3^; Table 2). Galectin-1 was associated with increased risk of CKD after age and sex adjustment (HR 1.18 per SD increase; 95% CI 1.09, 1.28; *p* = 4.9 × 10^−5^). However, while the association remained significant after adjustment for known risk factors, the association was strongly attenuated and not significant after adjustment for baseline eGFR (*p* = 0.84; Table 2). Furthermore, galectin-1 was associated with increased risk of type 2 diabetes after age and sex adjustment (HR 1.30 per SD increase; 95% CI 1.19, 1.43; *p* = 1.4 × 10^−8^; Table 2). The association remained significant but attenuated after adjustment for established risk factors (*p* = 0.021; Table 2). Galectin-1 was not associated with any of the secondary outcomes examined including coronary artery disease and mortality outcomes (ESM Table 2). Including galectin-1 in addition to established risk factors did not improve model discrimination of CKD or type 2 diabetes. For CKD, the addition of galectin-1 marginally increased the C statistic from 0.7346 to 0.7353 (*p* = 0.003) in addition to established risk factors (multivariable model adjusting for age, sex, use of anti-hypertensive treatment, systolic blood pressure, BMI, smoking status, C-reactive protein, fasting blood glucose, prevalent diabetes mellitus and baseline eGFR; Table 2), and the cNRI for galectin-1 was 0.1742 (*p* = 2.0 × 10^−4^). There was no improvement in cNRI for galectin-1 when further including baseline eGFR. The C statistic for models with and without galectin-1 in addition to established risk factors for type 2 diabetes did not differ, and the cNRI for galectin-1 was 0.0281 (*p* = 0.55).

**Table 2:** Longitudinal associations of galectin-1 measured at baseline examination in the MDCS-CC with eGFR, CKD and type 2 diabetes

Outcome	Quartiles of galectin-1 levels				Per SD increase	*p* value
	Q1	Q2	Q3	Q4		
eGFR						
Number of participants	591	588	590	589		
Baseline eGFR (ml min^−1^ [1.73 m]^−2^)	96.3 (12.0)	91.2 (11.9)	88.6 (12.3)	86.0 (12.7)		
Follow-up eGFR (ml min^−1^ [1.73 m]^−2^)	70.6 (14.5)	67.6 (14.3)	64.6 (14.3)	63.0 (16.0)		
Absolute change in eGFR during follow-up (ml min^−1^ [1.73 m]^−2^)^a^	−25.7 (13.0)	−23.6 (12.9)	−24.0 (13.4)	−23.1 (14.2)	0.22 (−0.32, 0.76)	0.425
Mean annual change in eGFR (%)^b^	−1.59 (0.79)	−1.56 (0.86)	−1.64 (0.89)	−1.63 (1.03)	0.05 (0.02, 0.09)	5.6 × 10^−3^
CKD						
Number of participants	563 (126)	574 (156)	565 (205)	549 (211)		
Unadjusted HR (95% CI)	1.00 (ref)	1.33 (1.05, 1.68)	1.96 (1.57, 2.44)	2.09 (1.67, 2.60)	1.32 (1.23, 1.43)	6.2 × 10^−13^
Age- and sex-adjusted HR (95% CI)	1.00 (ref)	1.13 (0.89, 1.43)	1.55 (1.24, 1.94)	1.56 (1.24, 1.95)	1.18 (1.09, 1.28)	4.9 × 10^−5^
Multivariable-adjusted HR (95% CI)^c^	1.00 (ref)	1.15 (0.91, 1.46)	1.51 (1.21, 1.90)	1.43 (1.14, 1.80)	1.13 (1.04, 1.22)	3.1 × 10^−3^
Multivariable-adjusted HR (95% CI)^d^	1.00 (ref)	0.96 (0.76, 1.22)	1.13 (0.90, 1.43)	1.05 (0.83, 1.32)	0.99 (0.91, 1.08)	0.84
Type 2 diabetes						
Number of participants	830 (106)	836 (125)	827 (142)	741 (199)		
Unadjusted HR (95% CI)	1.00 (ref)	1.18 (0.91, 1.53)	1.36 (1.06, 1.75)	2.11 (1.67, 2.68)	1.35 (1.24, 1.48)	3.2 × 10^−11^
Age- and sex-adjusted HR (95% CI)	1.00 (ref)	1.15 (0.89, 1.49)	1.27 (0.98, 1.63)	1.89 (1.49, 2.41)	1.30 (1.19, 1.43)	1.4 × 10^−8^
Multivariable-adjusted HR (95% CI)^e^	1.00 (ref)	0.97 (0.74, 1.27)	1.02 (0.78, 1.32)	1.27 (0.98, 1.64)	1.12 (1.03, 1.23)	0.013
Multivariable-adjusted HR (95% CI)^f^	1.00 (ref)	0.95 (0.71, 1.26)	1.01 (0.77, 1.33)	1.26 (0.97, 1.65)	1.12 (1.02, 1.24)	0.021

eGFR data presented for quartiles of galectin-1 are mean (SD) or HR (95% CI), unless stated otherwise. The per SD increase denotes β coefficient from a linear regression model

^a^Absolute change in eGFR (follow-up eGFR minus baseline eGFR) per SD increase in baseline galectin-1 estimated using a linear regression model, adjusting for age, sex, use of anti-hypertensive treatment, systolic blood pressure, BMI, smoking status, C-reactive protein, fasting blood glucose, prevalent diabetes mellitus and baseline eGFR

^b^Annual change in eGFR ([(absolute change/baseline eGFR) × 100]/years of follow-up) per SD increase in galectin-1 estimated using a linear regression model, adjusting for age, sex, use of anti-hypertensive treatment, systolic blood pressure, BMI, smoking status, C-reactive protein, fasting blood glucose and prevalent diabetes mellitus

^c^HR from a Cox proportional hazards regression model with follow-up time (years) until re-examination 2007–2012 as the timescale and adjusting for age, sex, use of anti-hypertensive treatment, systolic blood pressure, BMI, smoking status, C-reactive protein and fasting blood glucose. Participants with diabetes mellitus and CKD at baseline examination were excluded from the analysis

^d^Model described in footnote c, with additional adjustment for baseline eGFR

^e^HR from a Cox proportional hazards regression model with follow-up time (years) until 31 December 2014 as the timescale and adjusting for age, sex, use of anti-hypertensive treatment, systolic blood pressure, BMI, smoking status, family history of diabetes, fasting blood glucose, C-reactive protein, HDL-cholesterol and triacylglycerols. Participants with diabetes mellitus at baseline examination were excluded from analysis

^f^Model described in footnote e with additional adjustment for baseline eGFR

Ref, reference quartile

### Galectin-1 and eGFR among subgroups of individuals with type 2 diabetes (ANDIS study)

Clinical characteristics of ANDIS participants with measured galectin-1 are shown in ESM Table 3. The mean levels of galectin-1 and eGFR across type 2 diabetes subgroups are shown in ESM Fig. 1. Galectin-1 levels significantly differed across subgroups after adjustment for age and sex (*p* = 9.76 × 10^−4^) but not after additional adjustment for BMI (all *p* > 0.05). The SIRD group had significantly higher galectin-1 levels compared with both SIDD and MARD groups but not compared with the MOD group. In a linear regression model adjusting for age, sex, BMI, HOMA2-IR, HOMA2-B and HbA_1c_, there was a significant inverse association between galectin-1 levels and eGFR among individuals with type 2 diabetes at the time of diagnosis (*p* = 0.001). This association was driven primarily by a strong inverse association between galectin-1 and eGFR among the SIRD group (β coefficient = −45.60, SEM = 15.01; *p* = 0.005) and the MARD group (β coefficient = −47.37, SEM = 15.56; *p* = 0.004), but the difference between subgroups was not statistically significant. Scatter plots and linear regression models showing the correlation between galectin-1 and eGFR in type 2 diabetes subgroups are presented in ESM Fig. 2 and ESM Table 4.

### MR analysis of CKD, eGFR and type 2 diabetes

**Fig. 3:**
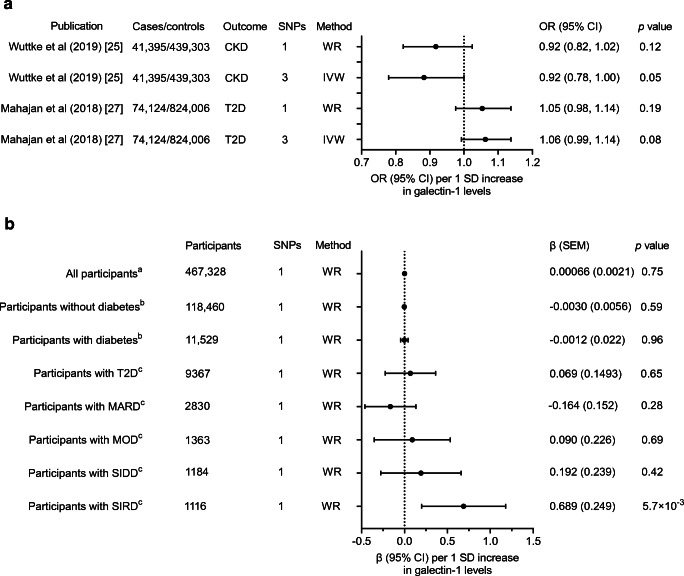
Two-sample MR analyses for the association of genetically predicted serum galectin-1 levels with (**a**) CKD and type 2 diabetes and (**b**) creatinine-based eGFR, overall and stratified by diabetes mellitus status. ^a^Wuttke et al (2019) [25]; ^b^Pattaro et al (2016) [26]; ^c^ANDIS cohort. T2D, type 2 diabetes; WR, Wald ratio

The main results from the MR analyses are shown in Fig. 3. The sentinel SNP (rs7285699) did not present a significant effect of galectin-1 levels on odds of CKD (Wald ratio OR 0.92; 95% CI 0.82, 1.02; *p* = 0.12; Fig. 3a) in the CKDGen Consortium Study. Including two additional variants in the MR analysis reduced the *p* value while maintaining the effect size but did not demonstrate any significant protective effect on CKD (IVW OR 0.88; 95% CI 0.78, 1.00; *p* = 0.0499). There was no association with eGFR (*p* = 0.75). MR analyses of the effect of galectin-1 among the four subgroups of type 2 diabetes showed that, among the SIRD group, genetically elevated levels of galectin-1 were positively associated with eGFR (*p* = 5.7 × 10^−3^; Fig. 3b). We observed no significant effect of genetically elevated levels of galectin-1 on odds of type 2 diabetes using either the sentinel SNP (Wald ratio OR 1.05; 95% CI 0.98, 1.14; *p* = 0.19; Fig. 3a) or three variants within the *LGALS1* locus (IVW OR 1.06; 95% CI 0.99, 1.14; *p* = 0.082) in DIAGRAM.

## Discussion

This is to the best of our knowledge the first large, population-based cohort study examining the longitudinal association of galectin-1 with CKD and type 2 diabetes. We demonstrate that cross-sectionally circulating galectin-1 levels are strongly inversely associated with eGFR as a measure of kidney function and several quantitative traits defining the metabolic syndrome. Furthermore, we demonstrate associations with incidence of CKD and type 2 diabetes independently of established risk factors. However, after adjusting for baseline eGFR, the association with incident CKD completely vanished. In line with this observation, we observed a non-significant direction of effect towards a causal protective effect of galectin-1 on odds of CKD in CKDGen and for higher odds of type 2 diabetes in DIAGRAM. We also show that individuals with type 2 diabetes in the SIRD cluster in the ANDIS study have higher galectin-1 levels compared with other diabetes clusters and that individuals in the SIRD group with higher galectin-1 have lower eGFR. Nevertheless, MR analyses suggest a causal protective role of galectin-1 in the SIRD cluster of the ANDIS study.

Higher circulating galectin-1 levels at baseline were strongly associated with measures of kidney function, in line with previous reports [8, 9]. Earlier studies have proposed a role for galectin-1 in kidney disease but were mainly based on smaller experimental studies [7, 8, 29, 30]. The relationship between obesity, type 2 diabetes and declined renal function is well known [31–33], although the causal pathways are not fully established [34]. In agreement with earlier epidemiological studies, BMI and related clinical markers of the metabolic syndrome correlated with circulating galectin-1 levels in our study [17, 35]. Conversely, we have previously reported that there is an inverse relationship between galectin-1 and type 2 diabetes when adjusting for BMI [17]. A study on pregnant women also reported that participants develop gestational diabetes when the adaptive increase of galectin-1 is impaired during pregnancy [36]. Similar to previous findings, participants with prevalent diabetes mellitus at baseline in our study had lower galectin-1 levels after adjustment for BMI. Further, the prospective analyses suggested a positive association between galectin-1 levels at baseline and incidence of type 2 diabetes, independently of established risk factors. However, MR results did not provide evidence for a causal effect of galectin-1 levels on incidence of type 2 diabetes. Our results indicate that galectin-1 levels may not directly affect type 2 diabetes risk but do not exclude a mediating role affecting insulin resistance, e.g. through lifestyle. In fact, earlier studies have shown that changes in energy intake alter galectin-1 gene expression in human subcutaneous adipose tissue [16, 37].

The strong cross-sectional association of galectin-1 with BMI, insulin resistance and markers of renal function could suggest a joint role for galectin-1 in these processes. Therefore, and due to our MR results indicating a possible protective effect on CKD, we examined the association between galectin-1 and eGFR among individuals with newly diagnosed type 2 diabetes in the ANDIS cohort, particularly those with SIRD previously reported to have increased risk of diabetic kidney disease [3]. We found that galectin-1 levels were higher in the SIRD group (compared with other type 2 diabetes subgroups), and, among individuals with SIRD, higher galectin-1 levels were cross-sectionally associated with lower eGFR. However, it is well known that significant observational associations never testify to causality or directionality of the potential causal effects in cross-sectional analyses. As a testimony of this, our MR analyses suggest a causal connection between genetically elevated galectin-1 levels and better kidney function in the SIRD group. This is also in concordance with our MR results in CKDGen, where there were no significant results, but the direction of effect gravitated towards lower odds of CKD and higher odds of type 2 diabetes in DIAGRAM.

It may appear counterintuitive that genetically elevated galectin-1 levels could have a protective effect on kidney function, while cross-sectional associations are seen between higher galectin-1 levels and lower eGFR. However, higher circulating galectin-1 levels could indicate a protective response to renal tissue damage. In line with this observation, our MR analysis suggests that individuals with genetically higher galectin-1 levels may experience a renal protective effect. Indeed, a recent study demonstrated renal protective effects of galectin-1 in rats [10]. Previous studies have also reported that galectin-1 levels increase in eyes of individuals with diabetes after accumulation of d-AGEs, as a direct response to the induced inflammation [38].

Our study has some limitations to consider. Galectin-1 was measured at baseline and analysed years after the initial sampling; however, we have tested the stability of galectin-1 in five repeated freeze–thaw cycles of biobanked samples without significant changes in galectin-1 concentration (data not shown). Furthermore, galectin-1 levels measured in this cohort were comparable with those in our recent study [17]. Another limitation is that diabetes type was not specified for 46% of the incident cases in the MDCS-CC. These cases were considered to be type 2 diabetes given that the minimum age at diagnosis was 48 years. Further, the MDCS-CC is a single-centre observational study, and the associations reported need confirmation in other populations and ethnicities. Finally, we used two-sample MR analyses for causal inference. We explored the causal direction of effects using a two-sample MR approach with a single genome-wide significant SNP as the genetic instrument. However, MR analyses of CKD and type 2 diabetes were not statistically significant, and it is possible that including additional genetic instruments to increase the statistical power of the genetic instrument could provide more robust answers to the question of causality. We therefore performed a stepwise conditional analysis to identify two additional variants; however, these variants were not associated with galectin-1 on a genome-wide significant level and only marginally increased the strength of the instrument. The association with CKD presented a *p* < 0.05, which was not considered significant due to the Bonferroni adjustment. Adjustment for multiplicity in hypothesis-driven studies has been questioned [39], and a type 2 error cannot be ruled out. In addition, weak instruments tend to bias estimated causal effects towards the observational association. Thus, additional GWAS on galectin-1 using larger sample sizes would be useful for identifying additional variants and allowing for a more robust genetic instrument.

Our MR analysis indicates a potential renal protective effect of galectin-1 based on our observation that genetically elevated levels of galectin-1 are associated with higher eGFR in an independent study population of individuals with type 2 diabetes at high risk of diabetic nephropathy. This observation is supported by a non-statistically significant direction of effect in CKDGen that cannot rule out any potential protective effect of galectin-1 on CKD in the MR analysis. Notably, no effect of galectin-1 on eGFR was observed in the MR analysis in the MARD cluster in ANDIS although there was an association between galectin-1 and eGFR in MARD, indicating a potentially unique role of galectin-1 in individuals with SIRD. An isolated finding in the ANDIS SIRD cluster is, however, difficult to interpret, since spurious findings could be found in this stratified analysis. Individuals with SIRD were selected on two factors related to galectin-1, both type 2 diabetes and propensity for kidney disease; thus, collider bias could potentially be introduced in the MR analyses conducted within the ANDIS cohort.

In conclusion, galectin-1 shows a strong cross-sectional association with decreased kidney function, but two-sample MR analyses suggest a causal protective effect of galectin-1 on kidney function among participants with type 2 diabetes at high risk of diabetic nephropathy. Future studies are needed to explore the mechanisms by which galectin-1 affects kidney function and whether it could be a useful target for improving kidney function among individuals with type 2 diabetes.

## Supplementary information

ESM (PDF 219 kb)

## Data Availability

The datasets generated and/or analysed during the current study are not publicly available due to restrictions in the ethical permission, but the data can be accessed through the corresponding author upon reasonable request and with permission of the Malmö Diet and Cancer Study or the ANDIS Steering Committee.

## Abbreviations

ANDIS: All New Diabetics In Scania
CKD: Chronic kidney disease
CKDGen: Chronic Kidney Disease Genetics
cNRI: Category-free net reclassification improvement
DIAGRAM: DIAbetes Genetics Replication And Meta-analysis
GWAS: Genome-wide association study
hsCRP: High-sensitivity C-reactive protein
IVW: Inverse variance-weighted
MARD: Mild age-related diabetes
MDCS: Malmö Diet and Cancer Study
MDCS-CC: Malmö Diet and Cancer Study–Cardiovascular Cohort
MOD: Mild obesity-related diabetes
MR: Mendelian randomisation
SIDD: Severe insulin-deficient diabetes
SIRD: Severe insulin-resistant diabetes
