# Supplementary material for: The role of circulating galectin-1 in type 2 diabetes and chronic kidney disease: evidence from cross-sectional, longitudinal and Mendelian randomisation analyses
Source: Diabetologia. 2021 Nov 7;65(1):128–39. doi: 10.1007/s00125-021-05594-1 (PMC8660752; doi:10.1007/s00125-021-05594-1)
Supplement: Supplementary file 1 — (PDF 219 kb) [file 125_2021_5594_MOESM1_ESM.pdf]

Electronic Supplementary Material (ESM)

ESM Methods

MDCS-CC baseline measurements

Information on age and sex were extracted from the participants' Swedish personal identification number. Smoking status (never, former, current), educational level and use of anti-hypertensive treatment and lipid-lowering drugs was self-reported in the baseline questionnaire. All blood samples were donated at baseline after an overnight fast and direct measurements included HbA_{1c} (mmol/mol and %), triglycerides (mmol/l), and HDL (mmol/l). LDL (mmol/l) was estimated using Friedewald's formula. Blood samples stored at -80ºC were used for analyses of high-sensitivity C-reactive protein (hsCRP) levels using the Tina-quant® CRP latex high sensitive assay (Roche Diagnostics, Basel, Switzerland) on an ADIVA® 1650 Chemistry System (Bayer Healthcare, New York, USA). Cystatin C levels were measured using a particle-enhanced immunonephelometric assay (N Latex Cystatin, Dade Behring, Illinois, USA).

Type 2 diabetes ascertainment

Diabetes status at baseline and during follow-up and information on date of diabetes diagnosis was identified from seven registers as well as baseline and re-examination screenings of the MDCS and the Malmö Preventive Project, a population-based intervention study including some participants from the MDCS. The National Diabetes Register and the regional Diabetes 2000 Register required a proven diagnosis by a physician at the hospital based on international standards for diagnosis (i.e. fasting plasma glucose concentration ≥7.0 mmol/l measured twice). For cases not diagnosed at a hospital, the local HbA_{1c} register from the Department of Clinical Chemistry, Skåne University Hospital, Malmö, Sweden, was used.

Other registries used to identify diabetes cases included the National Patient Register, the Swedish Cause of Death Register (ICD-10 codes E10-E14 and O244-O249), and the Prescribed Drug Register (ATC code A10). The different sources of case ascertainment were overlapping. For analyses of incident diabetes, all subjects with prevalent diabetes mellitus (regardless of type) were excluded. Subjects with incident diabetes type specified as other than type 2 diabetes (type 1 diabetes, latent autoimmune diabetes in adults (LADA), secondary diabetes or other) were censored at their date of diagnosis and not included as incident type 2 diabetes cases.

Secondary outcomes

The study participants were followed for incident coronary artery disease (CAD) through record linkage using their Swedish personal identification number with the previously validated Swedish Hospital Discharge Register, the Swedish Cause of Death Register, and the Swedish Coronary Angiography and Angioplasty Registry. CAD was defined as coronary revascularisation, fatal or nonfatal myocardial infarction, or death due to ischaemic heart disease. Information on all-cause and cause-specific mortality was retrieved from the Swedish National Cause of Death Registry.

Genotyping quality control

The quality control (QC) procedures in the MDCS-CC have been presented in previously published papers using this data [1]. Individual level QC was performed by removing individuals with a call rate of <0.95, an inbreeding coefficient of >3 SD away from the mean, discordance between inferred and reported sex, duplicate samples, a second-degree relatedness or higher within the sample based on identify by decent sharing calculations, or deviating from the common population structure in the MDCS-CC (exceeding 8 σ on first two

principal components). Marker level QC was performed by filtering out variants if they had a call rate <95%, minor allele frequency of <0.01, variants on sex chromosomes, mitochondrial DNA, and variants showing an extreme deviation from Hardy-Weinberg equilibrium ($p<1 \times 10^{-6}$). After QC and imputation using the 1000 Genomes (1000G) Phase 1 version 3 reference panel 21,575,257 variants were left for genome-wide analysis in 4086 participants with measured serum galectin-1 levels.

Construction of the multi-SNP instrument.

In order to improve the statistical power of our genetic instrument we considered inclusion of additional *cis* variants in the genome-wide significant *LGALS1* locus. The sentinel SNP was defined as the SNP with the lowest p-value (rs7285699). We used GCTA-COJO to perform conditional and joint multiple-SNP analysis of the genome-wide significant locus (+/- 300 kb from *LGALS1*). A stepwise selection of additional variants was performed based on a conditional *p* value threshold of 0.01. A less stringent p-value threshold was selected to not miss informative tag SNPs due to their mild LD with selected variants. In total three variants were retained and the joint effect estimates, standard errors and *p* values were calculated and used in a fixed effect inverse variance weighted analysis to assess the causal effect of galectin-1 on CKD and T2D (ESM Table 1).

References

[1] Schulz CA, Christensson A, Ericson U, et al. (2017) High Level of Fasting Plasma Proenkephalin-A Predicts Deterioration of Kidney Function and Incidence of CKD. J Am Soc Nephrol 28(1): 291-303. 10.1681/ASN.2015101177

ESM Table 1 - Description of SNPs included in the genetic instrument for galectin-1.

SNP ID	Chr	Position	Effect allele	Freq	Beta	SEM	*p* value	$Beta_{joint}$	SEM_{joint}	$p\ value_{joint}$
rs7285699	22	38066556	C	0.406	-0.16573	0.024747	2.13E-11	-0.15262	0.025257	1.52E-09
rs2281096	22	37960825	A	0.622	0.096017	0.025086	0.00013	0.071878	0.025497	0.004816
rs139963997	22	38282558	A	0.0158	-0.31438	0.090466	0.00051	-0.31120	0.090615	0.000594

Abbreviations: Chr – chromosome; Freq - allele frequency; SEM - standard error of the mean Effect sizes derived in a sample of 4086 subjects of the MDCS-CC. Joint effect estimates are from the conditional analysis with stepwise exclusion of variants using a conditional *p* value threshold of 0.01.

ESM Table 2 - Longitudinal association between baseline galectin-1 and secondary outcomes.

**Outcome**	**Quartiles of galectin-1 levels**					
	**Q1**	**Q2**	**Q3**	**Q4**	**Per SD increase**	***p* value**
Coronary artery disease						
Number of subjects (cases)	944 (69)	936 (86)	938 (89)	930 (101)		
Unadjusted HR	3.7 (2.9-4.6)	4.7 (3.8-5.8)	4.8 (3.9-6.0)	5.8 (4.8-7.0)		
Age- and sex-adjusted HR	1.00 (ref)	1.19 (0.87-1.63)	1.13 (0.83-1.55)	1.27 (0.93-1.73)	1.09 (0.98-1.22)	0.12
Multivariable adjusted HR[1]	1.00 (ref)	1.03 (0.75-1.42)	0.96 (0.70-1.33)	0.92 (0.67-1.27)	0.97 (0.87-1.09)	0.65
All-cause mortality						
Number of subjects (cases)	854 (165)	862 (191)	850 (191)	818 (243)		
Unadjusted HR	9.4 (8.1-11.0)	11.0 (9.5-12.6)	11.1 (9.6-12.8)	15.1 (13.3-17.1)		
Age- and sex-adjusted HR	1.00 (ref)	1.04 (0.84-1.28)	0.93 (0.76-1.15)	1.17 (0.95-1.43)	1.04 (0.97-1.12)	0.27
Multivariable adjusted HR[2]	1.00 (ref)	1.01 (0.82-1.25)	0.89 (0.72-1.10)	1.09 (0.88-1.34)	1.01 (0.94-1.09)	0.75
CVD mortality						
Number of subjects (cases)	854 (32)	862 (46)	850 (43)	818 (72)		
Unadjusted HR	1.8 (1.3-2.6)	2.6 (2.0-3.5)	2.5 (1.9-3.4)	4.5 (3.5-5.6)		
Age- and sex-adjusted HR	1.00 (ref)	1.21 (0.77-1.90)	0.97 (0.61-1.53)	1.57 (1.03-2.39)	1.16 (1.00-1.34)	0.057
Multivariable-adjusted HR[2]	1.00 (ref)	1.12 (0.71-1.77)	0.85 (0.53-1.35)	1.27 (0.82-1.95)	1.07 (0.91-1.25)	0.43
Cancer mortality						
Number of subjects (cases)	854 (82)	862 (77)	850 (85)	818 (97)		
Unadjusted HR	4.7 (3.8-5.8)	4.4 (3.5-5.5)	4.9 (4.0-6.1)	6.0 (4.9-7.3)		
Age- and sex-adjusted HR	1.00 (ref)	0.88 (0.64-1.20)	0.91 (0.67-1.24)	1.05 (0.78-1.42)	1.04 (0.93-1.16)	0.54
Multivariable-adjusted HR[2]	1.00 (ref)	0.87 (0.64-1.19)	0.89 (0.65-1.21)	1.00 (0.74-1.37)	1.02 (0.91-1.14)	0.79
Other cause mortality						
Number of subjects (cases)	854 (51)	862 (68)	850 (63)	818 (74)		
Unadjusted HR	2.9 (2.2-3.8)	3.9 (3.1-4.9)	3.7 (2.9-4.7)	4.6 (3.7-5.8)		
Age- and sex-adjusted HR	1.00 (ref)	1.16 (0.81-1.68)	0.95 (0.65-1.37)	1.09 (0.76-1.56)	0.97 (0.86-1.11)	0.67
Multivariable adjusted HR[2]	1.00 (re)	1.16 (0.80-1.67)	0.94 (0.65-1.37)	1.10 (0.75-1.59)	0.97 (0.85-1.11)	0.70

HR and 95% CI for secondary outcomes according to quartiles of galectin-1 and per SD increment in galectin-1 levels among subjects in the MDCS-CC.
Abbreviations: CVD – cardiovascular disease
1 Adjusted for age, sex, use of antihypertensive medication, systolic blood pressure, BMI, current smoking status, and fasting concentrations of HDL-cholesterol, LDL-cholesterol, hsCRP and prevalent diabetes mellitus. Analyses for incident coronary artery disease excluded subjects with a history of cardiovascular events.
2 Adjusted for age, sex, use of antihypertensive medication, systolic blood pressure, BMI, current smoking status, and fasting concentrations of HDL-cholesterol, LDL-cholesterol, and hsCRP. Analyses for mortality outcomes excluded prevalent cases of cardiovascular disease, diabetes mellitus and malignant cancers.

ESM Table 3 – Baseline characteristics of ANDIS participants with measured galectin-1.

	**SIDD**	**SIRD**	**MOD**	**MARD**
Number of subjects	44	44	44	44
Galectin-1, AU	7.35 (0.27)	7.55 (0.20)	7.49 (0.18)	7.43 (0.17)
Age, years	59.1 (5.7)	66.0 (3.4)	50.6 (4.1)	67.7 (2.6)
Male sex, %	22 (50%)	22 (50%)	22 (50%)	22 (50%)
Prevalent CKD, %	0	15.9	2.3	0
Metformin, %	70.5	47.7	36.4	36.4
Insulin treatment, %	11.4	0	0	0
BMI, kg/m^2	28.9 (1.9)	34.1 (2.3)	35.2 (2.1)	28.1 (1.3)
Fasting glucose, mmol/l	11.4 (2.7)	7.3 (0.5)	7.6 (0.8)	7.1 (0.5)
C-peptide, nmol/l	1.05 (0.20)	2.18 (0.28)	1.35 (0.19)	1.04 (0.13)
HbA_{1c} at diagnosis, mmol/mol	98 (7)	50 (5)	52 (7)	48 (4)
HbA_{1c} at diagnosis, Mono-S	10.6 (1.6)	5.8 (1.4)	6.0 (1.5)	5.6 (1.3)
HOMA2-IR	3.1 (0.5)	5.4 (0.7)	3.4 (0.5)	2.6 (0.3)
eGFR , mL/min/1.73 m^2	97.5 (19.7)	80.7 (18.8)	96.3 (20.9)	86.3 (17.3)

Data presented as mean (SD). Abbreviations: AU – arbitrary units, CKD – chronic kidney disease, BMI – body mass index, HOMA2-IR – homeostatic model assessment for insulin resistance, eGFR – estimated glomerular filtration rate.

Mono-S was previously the standard method for HbA1c analysis in Sweden, normal range 3.9 - 5.3%

ESM Table 4 – Linear regression models for galectin-1 and eGFR in the different type 2 diabetes subgroups.

	**Sex-adjusted model**			**Multivariable-adjusted model**		
	**B**	**SEM**	***p* value**	**B**	**SEM**	***p* value**
MARD	-54.135	14.335	0.001	-47.369	15.585	0.004
MOD	-11.906	19.036	0.535	-18.937	20.132	0.354
SIDD	-14.349	11.808	0.231	-7.932	10.463	0.453
SIRD	-40.395	13.206	0.004	-45.598	15.012	0.005

Multivariable-adjusted model adjusted for sex, age at diabetes diagnose, body mass index, ln HOMA2-IR, ln HOMA2B and HbA1c.

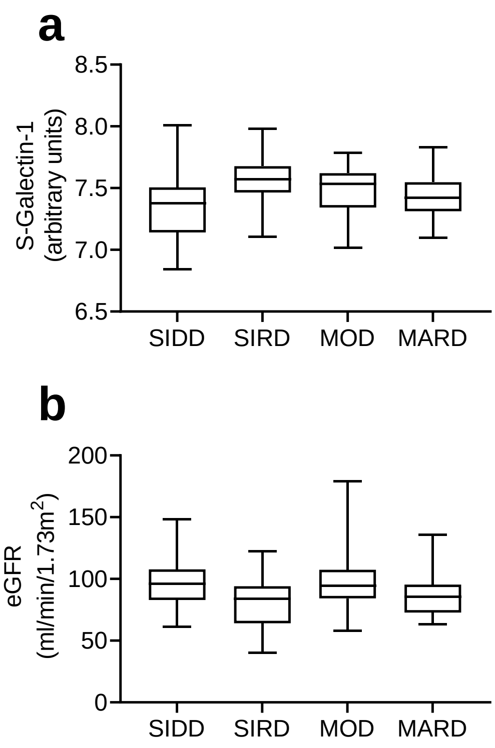

ESM Fig. 1 Box plots of plasma galectin-1 levels (a) and eGFR (b) across four clusters of type 2 diabetes patients in the ANDIS study.

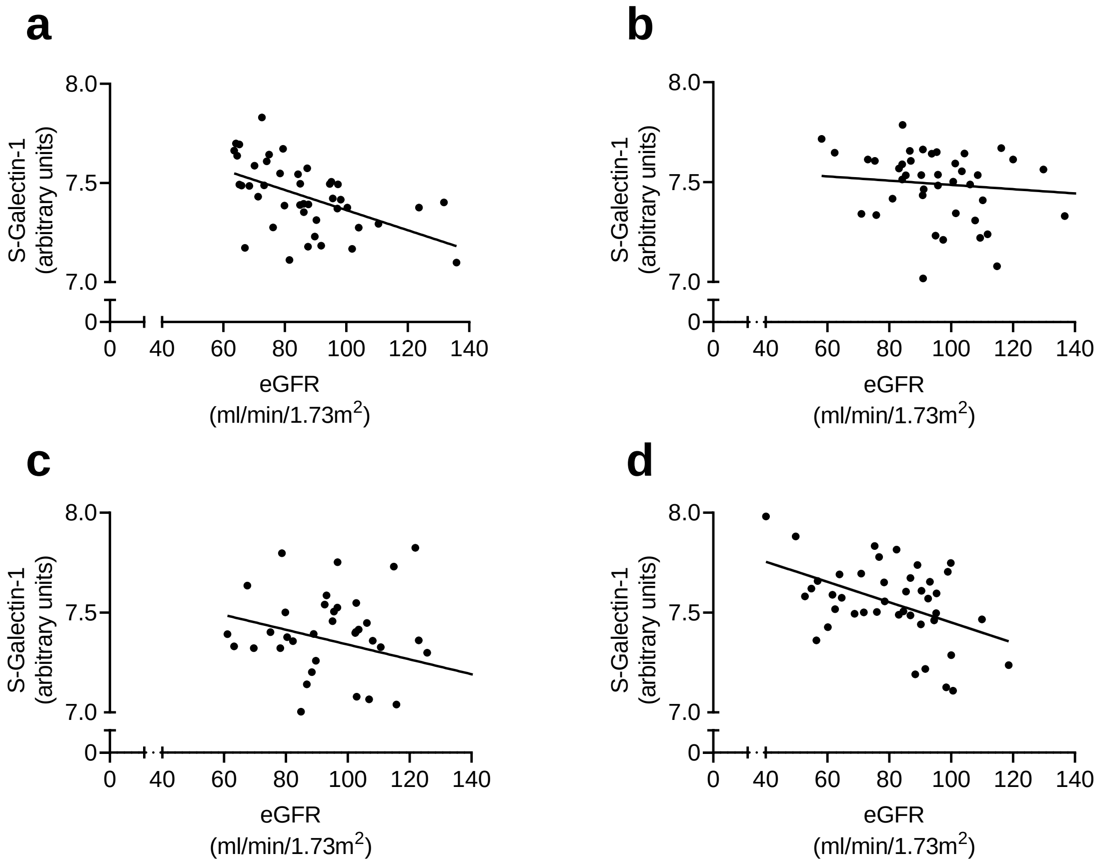

ESM Fig. 2 Scatter plots showing the correlation between plasma galectin-1 levels and eGFR at diabetes diagnosis among MARD (a), MOD (b), SIDD (c) and SIRD (d) type 2 diabetes clusters.
